# The Regulation of Calcium Homeostasis in T Lymphocytes

**DOI:** 10.3389/fimmu.2013.00432

**Published:** 2013-12-05

**Authors:** Gergely Toldi

**Affiliations:** ^1^First Department of Pediatrics, Semmelweis University, Budapest, Hungary

**Keywords:** calcium, T lymphocytes, lymphocyte activation, CRAC channel, CaV1 channels, autophagy, adenine nucleotides, mathematical modeling

Calcium is an important messenger in every cell type. Its intracellular level is regulated by finely tuned machinery responsible for calcium uptake, release, and intracellular storage. T cells are no exception in this regard. Pathways of calcium homeostasis participate in a number of cellular processes that determine short and long-term function of T lymphocytes. Over the recent year, an increasing number of calcium channels and transporters have been described that play a key role in balancing cytoplasmic calcium levels in T cells. Therapeutic strategies are now evolving based on the modulation of T-lymphocyte calcium homeostasis in order to combat immune-mediated disorders.

Submissions in this Research Topic include reviews on the ion channels that regulate calcium influx from the extracellular space in T cells (1, 2), either by conducting calcium ions or by modulating the membrane potential that provides the driving force for calcium influx (3, 4). The best characterized calcium channel in T cells is the calcium release-activated calcium (CRAC) channel, which is composed of ORAI and stromal interaction molecule (STIM) proteins. Several other channels may also mediate calcium influx directly in T cells including members of the transient receptor potential (TRP) family, P2X receptors, and voltage-gated calcium (Cav) channels. While the role of CRAC channels to T-cell function is well described by findings in ORAI1 and STIM1-deficient patients and mice (5, 6), the contributions of TRP, Cav1, and P2X receptor channels remain to be more clearly defined. These channels could contribute to calcium influx in specific T-cell subsets at distinct stages of T-cell development or following stimuli other than T-cell receptor (TCR) engagement.

Special attention is given to the emerging roles of Cav1 channels and their integration with other channels to generate a specific calcium signature in T lymphocytes (7, 8). The relationships between STIM, ORAI, and Cav1 will be discussed. For instance, STIM was shown to be a negative regulator of Cav1 signaling (9). Furthermore, the involvement of Cav1 channels as players in human disease will also be explored. In a recent study, a mixture of Cav1.2 and Cav1.3 specific antisense oligodeoxynucleotides strongly impaired the TCR-dependent increase of cytoplasmic calcium level and cytokine production in Th2 cells without any effect on Th1 cells, thus protecting mice against the development of asthma (10). These findings suggest that these channels may represent an interesting new approach in the treatment of allergic diseases.

The role of autophagy, a pathway for intracellular degradation in calcium homeostasis in T cells will also be reviewed (11). Autophagy regulates calcium signaling by developmentally maintaining the homeostasis of the ER (12).

Second messengers derived from the adenine dinucleotides, nicotinamide adenine dinucleotide (NAD), and nicotinamide adenine dinucleotide phosphate (NADP) have also been implicated in T-cell calcium signaling (13). Nicotinic acid adenine dinucleotide phosphate (NAADP) acts as a very early second messenger upon TCR/CD3 engagement, while cyclic ADP-ribose (cADPR) is mainly involved in sustained partial depletion of the endoplasmic reticulum by stimulating calcium release via ryanodine receptors. Finally, adenosine diphosphoribose (ADPR), a breakdown product of both NAD and cADPR activates the TRPM2 cation channel, thereby facilitating calcium (and sodium) entry into T cells. Receptor-mediated formation, metabolism, and mode of action of these novel second messengers in T lymphocytes will be reviewed. Their involvement in immune regulation also makes these pathways suitable targets for therapeutic intervention. The NAADP antagonist BZ194 has recently been shown to ameliorate the clinical course of transfer experimental autoimmune encephalitis, an animal model of multiple sclerosis (14).

While the crosstalk between calcium signaling and metabolic regulation in T cells is relatively poorly understood, one of the contributions highlights where such interactions occur (15). Calcium is known not only to mediate T-cell activation but it also modulates the unique metabolic changes that occur in distinct T-cell subsets and developmental stages. The crosstalk between mitochondrial metabolism, reactive oxygen species (ROS) generation, and CRAC channel activity will be highlighted.

In the course of lymphocyte activation, potassium channels maintain the driving force for sustained calcium influx from the extracellular milieu as they grant the efflux of potassium from the cytoplasm, thus conserving an electrochemical potential gradient between the intra- and extracellular spaces. There are two major types of potassium channels in T cells: the voltage-gated Kv1.3 and the calcium-activated IKCa1 channels. The relation between the calcium currents through CRAC channels and the efflux of potassium makes the proliferation and activation of lymphocytes sensitive to pharmacological modulation of Kv1.3 and IKCa1 channels (16), and provides an opportunity for targeted intervention that will also be discussed (17). Specific inhibition of these channels results in a diminished calcium influx in lymphocytes and a lower level of lymphocyte activation.

Finally, a quantitative mathematical model of T-lymphocyte calcium dynamics will be introduced that has been developed in order to establish a tool which helps to disentangle cause-effect relationships between ion fluxes and observed calcium time courses (18).
